# Predicting pathological complete response of neoadjuvant radiotherapy and targeted therapy for soft tissue sarcoma by whole-tumor texture analysis of multisequence MRI imaging

**DOI:** 10.1007/s00330-022-09362-6

**Published:** 2022-12-29

**Authors:** Lei Miao, Ying Cao, LiJing Zuo, HongTu Zhang, ChangYuan Guo, ZhaoYang Yang, Zhuo Shi, JiuMing Jiang, ShuLian Wang, YeXiong Li, YanMei Wang, LiZhi Xie, Meng Li, NingNing Lu

**Affiliations:** 1 Department of Radiology, National Cancer Center/National Clinical Research Center for Cancer/Cancer Hospital, Chinese Academy of Medical Sciences and Peking Union Medical College, 17 Panjiayuan Nanli, Chaoyang District, Beijing, 100021 China; 2 Department of Radiation Oncology, National Cancer Center/National Clinical Research Center for Cancer/Cancer Hospital, Chinese Academy of Medical Sciences and Peking Union Medical College, 17 Panjiayuan Nanli, Chaoyang District, Beijing, 100021 China; 3 Department of Pathology, National Cancer Center/National Clinical Research Center for Cancer/Cancer Hospital, Chinese Academy of Medical Sciences and Peking Union Medical College, 17 Panjiayuan Nanli, Chaoyang District, Beijing, 100021 China; 4 GE Healthcare China, Pudong New Town, Shanghai, China; 5 GE Healthcare, MR Research China, Beijing, 100176 China

**Keywords:** Sarcoma, Soft tissue, Neoadjuvant radiation therapy, Magnetic resonance imaging

## Abstract

**Objectives:**

To construct effective prediction models for neoadjuvant radiotherapy (RT) and targeted therapy based on whole-tumor texture analysis of multisequence MRI for soft tissue sarcoma (STS) patients.

**Methods:**

Thirty patients with STS of the extremities or trunk from a prospective phase II trial were enrolled for this analysis. All patients underwent pre- and post-neoadjuvant RT MRI examinations from which whole-tumor texture features were extracted, including T_1_-weighted with fat saturation and contrast enhancement (T_1_FSGd), T_2_-weighted with fat saturation (T_2_FS), and diffusion-weighted imaging (DWI) sequences and their corresponding apparent diffusion coefficient (ADC) maps. According to the postoperative pathological results, the patients were divided into pathological complete response (pCR) and non-pCR (N-pCR) groups. pCR was defined as less than 5% of residual tumor cells by postoperative pathology. Delta features were defined as the percentage change in a texture feature from pre- to post-neoadjuvant RT MRI. After data reduction and feature selection, logistic regression was used to build prediction models. ROC analysis was performed to assess the diagnostic performance.

**Results:**

Five of 30 patients (16.7%) achieved pCR. The Delta_Model (AUC 0.92) had a better predictive ability than the Pre_Model (AUC 0.78) and Post_Model (AUC 0.76) and was better than AJCC staging (AUC 0.52) and RECIST 1.1 criteria (AUC 0.52). The Combined_Model (pre, post, and delta features) had the best predictive performance (AUC 0.95).

**Conclusion:**

Whole-tumor texture analysis of multisequence MRI can well predict pCR status after neoadjuvant RT and targeted therapy in STS patients, with better performance than RECIST 1.1 and AJCC staging.

**Key points:**

• *MRI multisequence texture analysis could predict the efficacy of neoadjuvant RT and targeted therapy for STS patients.*

• *Texture features showed incremental value beyond routine clinical factors.*

• *The Combined_Model with features at multiple time points showed the best performance.*

**Supplementary Information:**

The online version contains supplementary material available at 10.1007/s00330-022-09362-6.

## Introduction

Soft tissue sarcoma (STS) is a relatively rare malignant tumor of mesenchymal origin, accounting for 1% of all malignant tumors [1]. In the modern era, limb-conserving wide resection in combination with neoadjuvant or adjuvant radiotherapy (RT) is the preferred treatment for nonmetastatic STS, with similar survival results as amputation but much better quality of life. With the results from the SR-2 randomized controlled trial, neoadjuvant RT is becoming the mainstream treatment modality due to lower radiation doses, smaller target volumes, and less irreversible late toxicities. However, the efficacy of preoperative RT alone is usually not satisfactory, with the vascular tortuosity and high proliferation commonly seen in STS as the main reasons of radiotherapy resistance. As reported, only approximately 8 to 10% of patients can achieve pathological complete response (pCR) after neoadjuvant RT [2–7]. Meanwhile, tyrosine kinase inhibitors (TKIs) can select appropriate targets, such as vascular endothelial growth factor receptor (VEGFR), platelet-derived growth factor receptor (PDGFR), and stem cell factor (SCF) receptor/c-kit, to block tumor-related signaling pathways, normalize the vascularization, and improve the oxygenation in tumor [8–10]. Thus, the combination of RT and targeted therapy could improve the pCR rate to approximately 20 to 30% [4–6]. It has been shown that patients who achieve pCR have a better long-term prognosis [11, 12]. Therefore, if the pCR status after neoadjuvant RT with targeted therapy for STS can be predicted by a noninvasive diagnostic modality with high accuracy, it will be of great clinical help.

However, the optimal efficacy evaluation criteria for neoadjuvant therapy for STS are still unclear. As the most commonly adopted clinical evaluation criteria for solid tumors, the Response Evaluation Criteria in Solid Tumors (RECIST) version 1.1 [13] does not perform well for STS, since the volume changes in STS are not necessarily related to efficacy due to underlying factors, including necrosis, intratumoral hemorrhage, and cystic changes [14–16]. Some studies have attempted to predict pCR with the AJCC stage at onset, but they have had little success [17–19]. The recently published Choi criteria [14, 20–22] provide new perspectives for early response evaluation by adding the signal or density change of enhanced scanning; however, it is still inconclusive for STSs on account of the limited numbers of reports and patients.

Magnetic resonance imaging (MRI) has high tissue resolution and is recognized as the most accurate modality for STS. Texture analysis (TA) can extract and calculate the grayscale changes in pixels or voxels from medical images and analyze quantitative image features to reflect the deep heterogeneity of tumor tissue [23]. It has shown certain value in the pathological grading and prognosis prediction of STS and differentiation of benign and malignant soft tissue masses [24–28]. From the perspective of treatment efficacy, some recent studies have shown good predictive performance [17, 29] in predicting the efficacy of neoadjuvant RT and/or chemotherapy in STS through the combination of radiomics features at multiple time points (delta radiomics).

However, there is no research about the prediction accuracy of MRI-based radiomics for patients receiving neoadjuvant RT and targeted therapy. Therefore, this study aimed to incorporate comprehensive MRI sequences to predict the pCR status of neoadjuvant RT and TKI in STS. We also compared the predictive performance of our models with that of the RECIST 1.1 criteria and AJCC stage.

## Materials and methods

### Patients

**Fig. 1:**
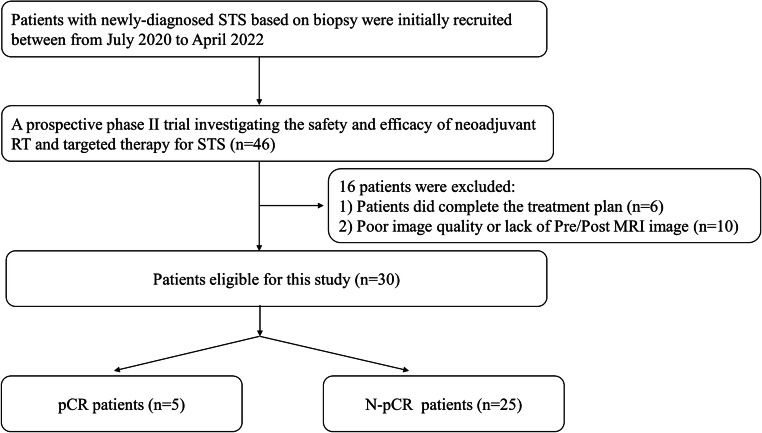
Flow diagram of the study cohort

Patients were enrolled from a prospective phase II trial investigating the safety and efficacy of neoadjuvant RT and targeted therapy (a multi-targeted TKI) for STS (*NCT05167994/ChiCTR2000033377, NCT05235100), with a prospective collection of MRI images before and after neoadjuvant RT. A total of 30 patients with STS admitted to the radiotherapy department of this research institution from July 2020 to April 2022 were enrolled in this study. The following inclusion criteria were applied: ① patients were older than 18 years; ② STS was pathologically proven, with an intermediate to high grade, maximum tumor size ≥ 5 cm, and deeply located tumor (≥ 2 items met); ③ neoadjuvant RT was required after multidisciplinary treatment (MDT) discussion; ④ no regional nodes or distant metastases were present; and ⑤ MRI was performed before and after neoadjuvant RT, and the image quality was good. The exclusion criteria were as follows: ① RT was not completed as planned; and ② MRI images before and/or after RT were missing or could not be compared. This study was performed in accordance with the Declaration of Helsinki, and informed consent was obtained from all subjects. The treatment and clinical efficacy assessment are detailed in the Supplementary Materials. The flow diagram of the study cohort is shown in Fig. 1.

### MR techniques

#### MRI scanning

All patients underwent MRI before and at the end of neoadjuvant RT. All MRI examinations were performed on a 3.0-T system (GE Discovery MR 750, General Electric Medical Systems) with an eight-element phased-array wrap-around surface coil. Both the field of view and the matrix matched the location and size of the tumor. The sequences included T_1_FSGd, T_2_FS, diffusion-weighted imaging (DWI), and their corresponding apparent diffusion coefficient (ADC) maps. The contrast agent used was gadoterate meglumine (Gd-DOTA), with a dose of 0.1 mmol/kg body weight, intravenously injected at a flow rate of 2.0 mL/s and then flushed with 20 mL of normal saline. The delayed images of enhanced scanning were collected 2 min after the injection of contrast agent. The details of all sequences are listed in Table S1.

### Imaging analysis

#### Tumor segmentation

The whole tumor was manually delineated slice-by-slice by two radiologists (with 3 years and 5 years of STS imaging diagnosis experience) using ITK-SNAP (version 2.2.0, www.itksnap.org) software and reviewed by an expert radiologist with 20 years of STS imaging diagnosis experience who was blinded to the clinical or pathological information. Regions of interest (ROIs) were delineated on each sequence independently. The scope of the ROI included the entire tumor and avoided peritumoral edema.

#### Feature extraction

**Fig. 2:**
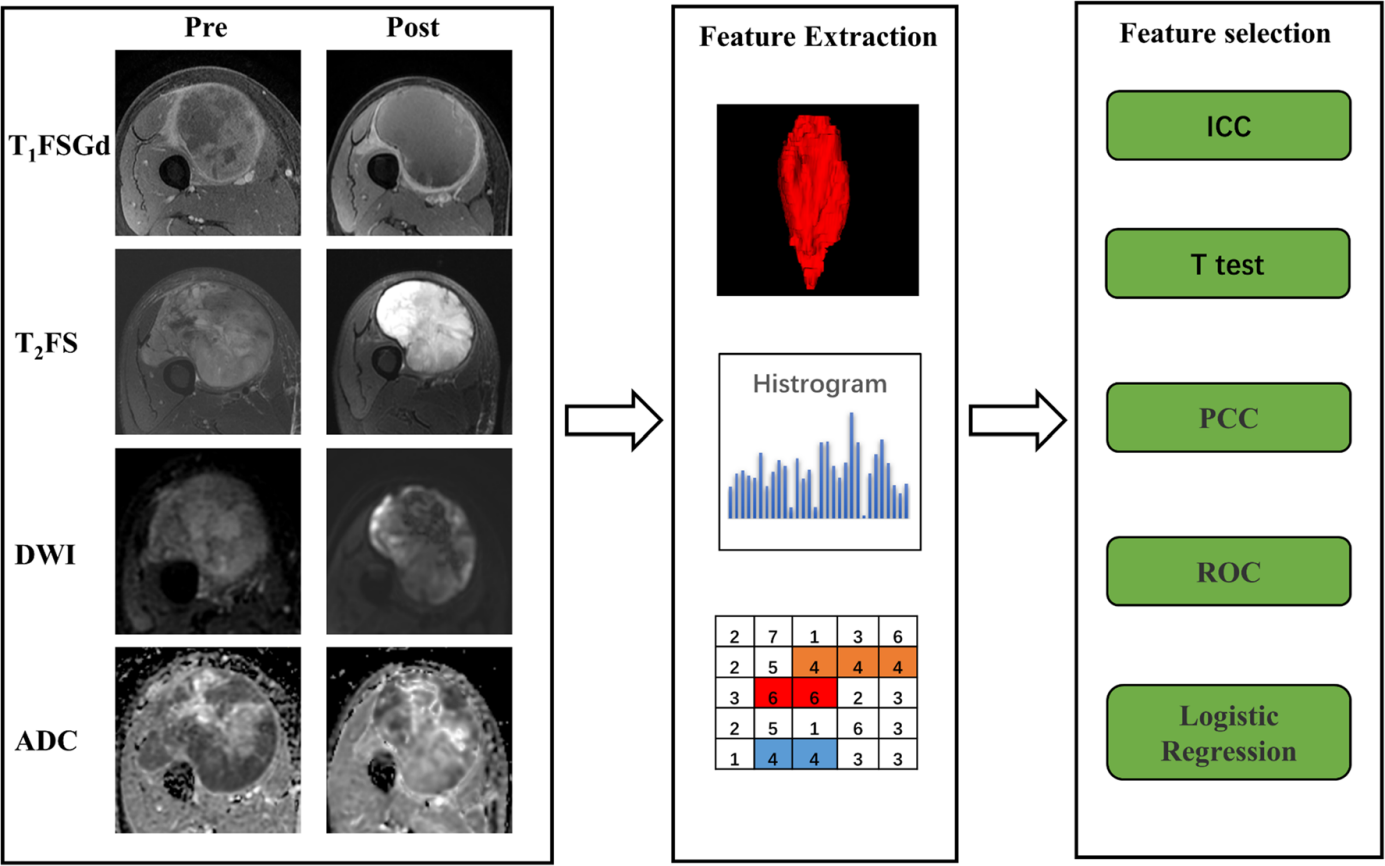
Workflow of texture feature analysis. Abbreviations: T_1_FSGd, T_1_-weighted with fat saturation and contrast enhancement; T_2_FS, T_2_-weighted with fat saturation; DWI, diffusion-weighted imaging; ADC, apparent diffusion coefficient; ICC, intraclass correlation coefficient; PCC, Pearson correlation coefficient; ROC, receiver operating characteristic

First, each MRI scan of each patient was normalized with *Z* scores to obtain a standard normal distribution of image intensities. Feature extraction was then performed on images of all sequences (T_1_FSGd, T_2_FS, DWI, ADC) for each patient. Each image had 107 texture features, including 14 shape features, 25 first-order features, 22 gray level co-occurrence matrix (GLCM) features, 16 gray level run length matrix (GLRLM) features, 16 gray level size zone matrix (GLSZM) features, and 14 gray level dependence matrix (GLDM) features. All texture features were obtained from open-source PyRadiomics (http://www.radiomics.io/pyradiomics.html) as recommended by IBSI [30]. Delta features were defined as the percentage change in a texture feature from pre- to post-neoadjuvant RT MRI. The calculation formula of the delta texture feature is as follows: Delta texture feature = (*X*_Pre_ − *X*_Post_) / *X*_Pre_, where *X*_Pre_ is the pre-neoadjuvant RT texture feature and *X*_Post_ is the post-neoadjuvant RT texture feature. The processes of tumor segmentation and feature extraction are shown in Fig. 2.

#### Feature selection and model construction

Data reduction and feature selection processes were performed to select the most relevant features for constructing the models. pCR status was defined as less than 5% of residual tumor cells by postoperative pathology. First, the texture features on pre, post, and delta images that were significantly different between the pCR group and the non-pCR (N-pCR) group were screened out. Second, the diagnostic efficacy of the above features was assessed by correlation tests and the area under the curve (AUC) method, and texture parameters with a high diagnostic efficacy and low correlation between groups (*r* < 0.8) were screened out. Finally, the statistically significant texture features were further screened out through logistic regression, and the Pre_Model, Post_Model, Delta_Model, and Combined_Model were established. In addition, the predictive ability of AJCC staging and RECIST 1.1 was assessed to compare the performance of various models.

### Statistical analysis

The data were analyzed using R software (version 3.6.1; http://www.Rproject.org) and MedCalc (ver. 10.3.0.0, MedCalc software). All data were assessed for a normal distribution using the Kolmogorov–Smirnov test. The *t* test was used to compare the differences between continuous variables, while the chi-square test or Fisher’s exact test was used to compare the differences between categorical variables. *p* values were corrected for multiple comparisons by Bonferroni. The intraclass correlation coefficient (ICC) was used to investigate interobserver agreement. The correlation between groups was judged by calculating the Pearson correlation coefficient, and *r* > 0.8 indicated a high correlation. Multivariate binary logistic regression analysis was conducted with a forward stepwise approach to select variables and construct models. The Mann–Whitney *U* test was used to evaluate the difference in the RadScore between the pCR group and the N-pCR group for each model. Receiver operating characteristic (ROC) curves were applied to evaluate the diagnostic performance. The Youden index was used to determine the optimal threshold, and the AUC, sensitivity, specificity, and accuracy were calculated. The DeLong method was used to compare the AUC values of all models. The Hosmer–Lemeshow test was used to assess the goodness-of-fit of the models. Decision curve analysis (DCA) was performed according to the method of Vickers et al [31]. DCA explored the benefit of different models by calculating the net benefit of each decision strategy at each threshold probability. In this study, a two-sided *p* value < 0.05 was considered statistically significant.

## Results

### Patient characteristics

Thirty patients (18 males, 12 females; mean age, 53.0 ± 15.3 years) were included in this study, of which 5 patients (16.7%) showed pCR on postoperative pathology. All clinical and pathological data are shown in Table 1.

**Table 1:** Clinical, pathological, and radiological data of the patients

Characteristic	All patients	pCR	N-pCR	*p* value
Sex				
Male	18 (60%)	5 (100%)	13 (52%)	0.066
Female	12 (40%)	0 (0%)	12 (48%)	
Age at diagnosis (y)	54 (22, 75)	67 (52, 75)	52 (22, 72)	0.037*
Size	10.71 ± 4.26 (cm)	10.10 ± 2.46 (cm)	10.84 ± 4.57 (cm)	0.731
Location				
Upper limb	4 (13.3%)	0 (0%)	4 (16%)	0.373
Lower limb	18 (60%)	3 (60%)	15 (60%)	
Trunk	8 (26.7%)	2 (40%)	6 (24%)	
T stage				
1	3 (10%)	0 (0%)	3 (12%)	0.867
2	9 (30%)	2 (40%)	7 (28%)	
3	14 (46.7%)	3 (60%)	11 (44%)	
4	4 (13.3%)	0 (0%)	4 (16%)	
Grade				
GX	3 (10%)	0 (0%)	3 (12%)	0.211
G1	2 (6.6%)	0 (0%)	2 (8%)	
G2	14 (46.7%)	2 (40%)	12 (48%)	
G3	11 (36.7%)	3 (60%)	8 (32%)	
AJCC stage				
I	5 (16.7%)	0 (0%)	5 (20%)	0.487
II	0 (0%)	0 (0%)	0 (0%)	
III	25 (83.3%)	5 (100%)	20 (80%)	
Histology				
Liposarcoma/myxoid liposarcoma	11 (36.7%)	2 (40%)	9 (36%)	0.76
Fibrosarcoma/Myxofibrosarcoma	8 (26.7%)	0 (0%)	8 (32%)	
Undifferentiated pleomorphic sarcoma	4 (13.3%)	2 (40%)	2 (8%)	
Inflammatory myofibroblastoma	2 (6.6%)	0 (0%)	2 (8%)	
Other sarcomas	5 (16.7%)	1 (20%)	4 (16%)	
Margin status post-operation				
Negative	27 (90%)	5 (100%)	22 (88%)	1.00
Positive	3 (10%)	0 (0%)	3 (12%)	
Treatment				
Radiotherapy + anlotinib	19 (63.3%)	3 (60%)	16 (64%)	0.978
Radiotherapy + apatinib	8 (26.7%)	2 (40%)	6 (24%)	
Radiotherapy	3 (10%)	0 (0%)	3 (12%)	
Clinical efficacy as per RECIST 1.1				
PR	13 (43.3%)	2 (40%)	11 (44%)	0.889
SD	17 (56.7%)	3 (60%)	14 (56%)	

Other sarcomas included pleomorphic rhabdomyosarcoma, round cell sarcoma, epithelioid sarcoma, myofibroblastoma, and synovial sarcoma. Grading was performed according to the French Federation of Cancer Centers Sarcoma Group (FNCLCC). *AJCC*, American Joint Committee on Cancer; *RECIST 1.1*, Response Evaluation Criteria in Solid Tumors version 1.1. *p < 0.05

### Feature selection

All parameters showed good interobserver agreement (ICC > 0.8). Among pre, post, and delta texture features, there were 4, 2, and 6 texture features, respectively, which were significantly different between the pCR and N-pCR groups (*p* < 0.05). Details of the texture features are shown in Table 2. Then, the Pearson correlation coefficient was calculated for the above features in their respective groups. An AUC comparison of the texture features with *r* > 0.8 between the two groups was carried out, and the texture features with lower AUCs were excluded. There were 3, 2, and 5 pre, post, and delta texture features, respectively, that met the requirements; see Tables S2–S4 for details.

**Table 2:** Remaining features after the significant difference test

Texture feature	pCR	N-pCR	*p* value	AUC (95%CI)
Pre_T_2__original_glrlm_GrayLevelVariance	15.28 ± 5.33	25.70 ± 14.22	0.012*	0.728 (0.536~0.920)
Pre_T_2__original_shape_Flatness	0.57 ± 0.20	0.44 ± 0.10	0.023*	0.776 (0.410~1)
Pre_T_2__original_firstorder_Range	954.48 (862.21, 1029.47)	1153.75 (1085.38, 1506.50)	0.024*	0.824 (0.674~0.974)
Pre_T_2__original_firstorder_Kurtosis	3.36 ± 0.48	4.06 ± 0.84	0.03*	0.736 (0.519~0.953)
Post_T_1__original_shape_Flatness	0.55 ± 0.15	0.44 ± 0.08	0.028*	0.760 (0.417~1)
Post_ADC_original_gldm_HighGrayLevelEmphasis	74.87 ± 10.42	95.89 ± 45.06	0.048*	0.712 (0.534~0.890)
Delta_T_1__original_glcm_ClusterShade	−3.59 (−181.53, 0.16)	0.77 (0.23, 1.16)	0.013*	0.856 (0.667~1)
Delta_T_1__original_shape_Sphericity	−0.08 (−0.14, −0.05)	0.02 (−0.06, 0.03)	0.037*	0.800 (0.590~1)
Delta_T_2__original_glszm_SizeZoneNonUniformity	−0.50 (−1.09, −0.09)	0.22 (−0.13, 0.55)	0.011*	0.864 (0.711~1)
Delta_T_2__original_firstorder_Energy	−0.35 (−0.48, −0.11)	0.30 (−0.05, 0.45)	0.042*	0.792 (0.634~0.950)
Delta_T_2__original_firstorder_Range	−0.40 ± 0.39	−0.05 ± 0.34	0.048*	0.736 (0.520~0.952)
Delta_T_2__original_firstorder_TotalEnergy	−0.35 (−1.21, −0.21)	0.17 (−0.40, 0.48)	0.048*	0.784 (0.614~0.953)

*AUC*, area under the receiver operating characteristic curve; *CI*, confidence interval; * < *p*＜0.05

### Model construction

The selected texture features were included in the multivariate logistic regression analysis, and texture features with *p* < 0.05 were excluded. The final texture feature of pre was Pre_T_2__original_shape_Flatness, the texture feature of post was Post_T_1__original_shape_Flatness, and the texture features of delta were Delta_T_1__original_glcm_ClusterShade and Delta_T_2__original_glszm_SizeZoneNonUniformity. The corresponding models were established by logistic regression. The Combined_Model was constructed by incorporating all the above texture features. The details of the multivariate logistic regression models are listed in Table 3.

**Table 3:** Model performance for predicting pCR status after neoadjuvant RT and targeted therapy in patients with STS

Model	AUC	95% CI	Sensitivity (%)	Specificity (%)	Accuracy (%)
Pre_Model	0.78	0.410~1	80	88	86.7
Pre_T_2__original_shape_Flatness					
Post_Model	0.76	0.417~1	80	88	83.3
Post_T_1__original_shape_Flatness					
Delta_Model	0.92	0.788~1	80	92	83.3
Delta_T_1__original_glcm_ClusterShade					
Delta_T_2__original_glszm_SizeZoneNonUniformity					
Combined_Model	0.95	0.874~1.000	100	84	88.7
Pre_T_2__original_shape_Flatness					
Post_T_1__original_shape_Flatness					
Delta_T_1__original_glcm_ClusterShade					
Delta_T_2__original_glszm_SizeZoneNonUniformity					
AJCC	0.52	0.305~0.743	100	16	56.7
RECIST 1.1	0.52	0.260~0.780	60	44	58.1

*AUC*, area under the receiver operating characteristic curve; *CI*, confidence interval; *AJCC*, American Joint Committee on Cancer; *RECIST 1.1*, Response Evaluation Criteria in Solid Tumors version 1.1

### Model comparison and evaluation

**Fig. 3:**
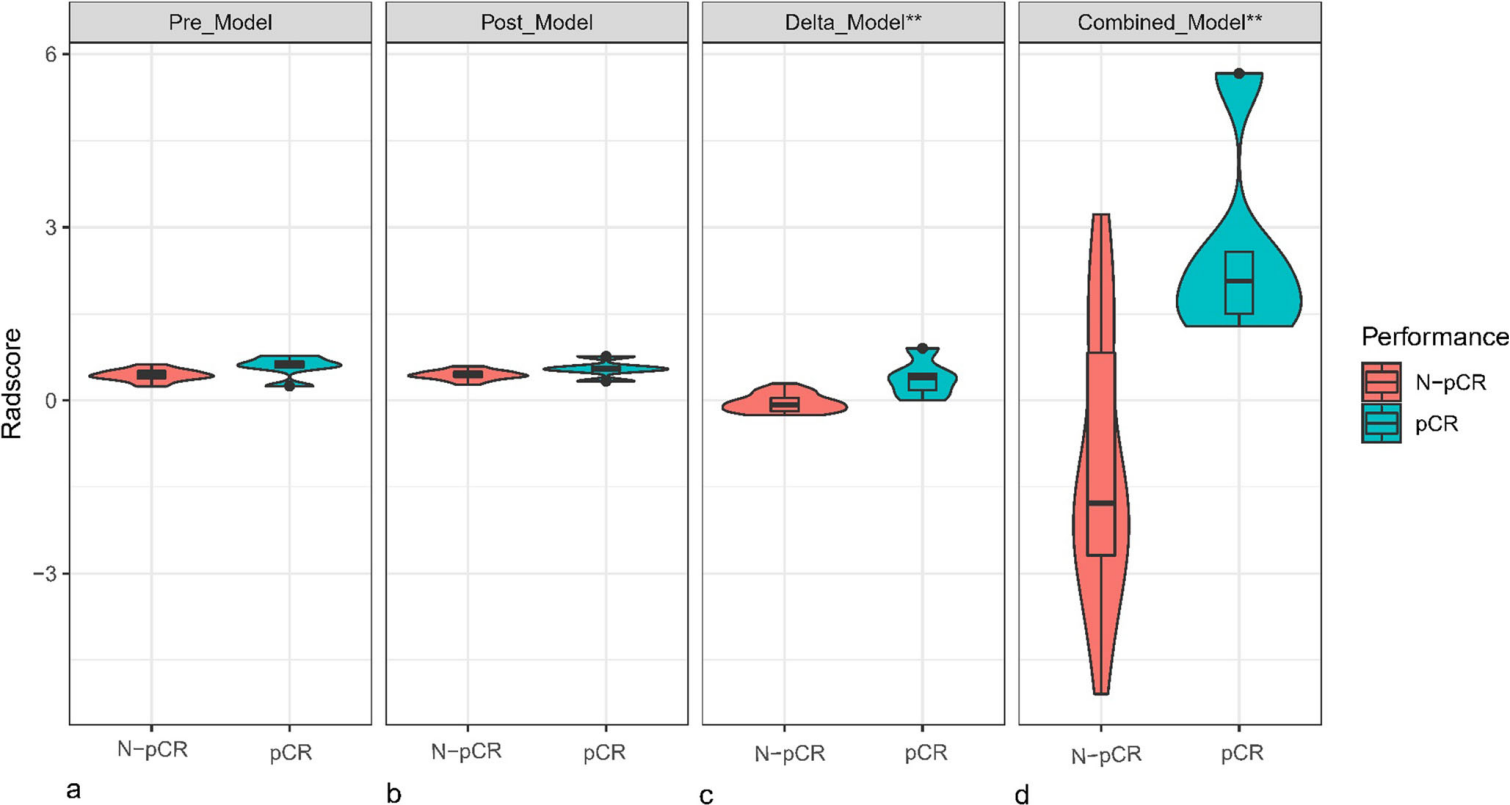
Distribution of the RadScore between the pCR and N-pCR groups in the four models. Density width indicates the frequency of the RadScore. The middle line in each box indicates the median value of the RadScore, and the lower and upper boundaries of the box indicate the first and third quartiles, respectively. Whiskers indicate the 95% confidence interval

**Fig. 4:**
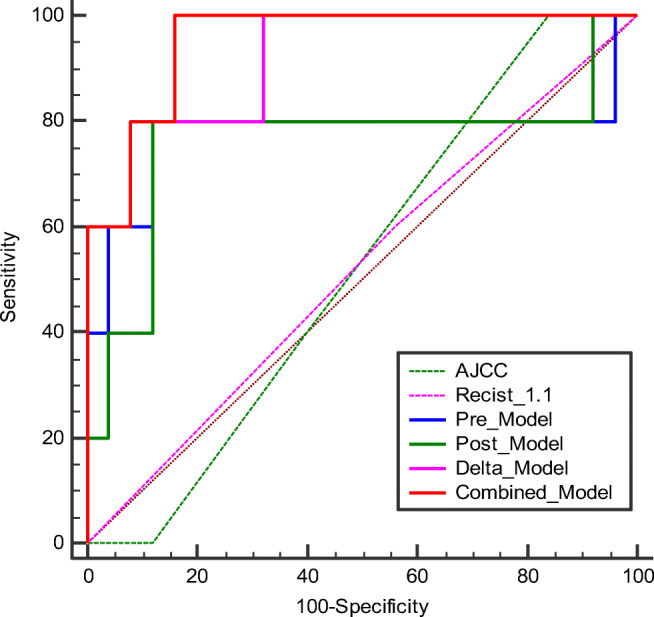
Comparison of the diagnostic performance of all models. AJCC, American Joint Committee on Cancer; RECIST 1.1, Response Evaluation Criteria in Solid Tumors version 1.1

**Fig. 5:**
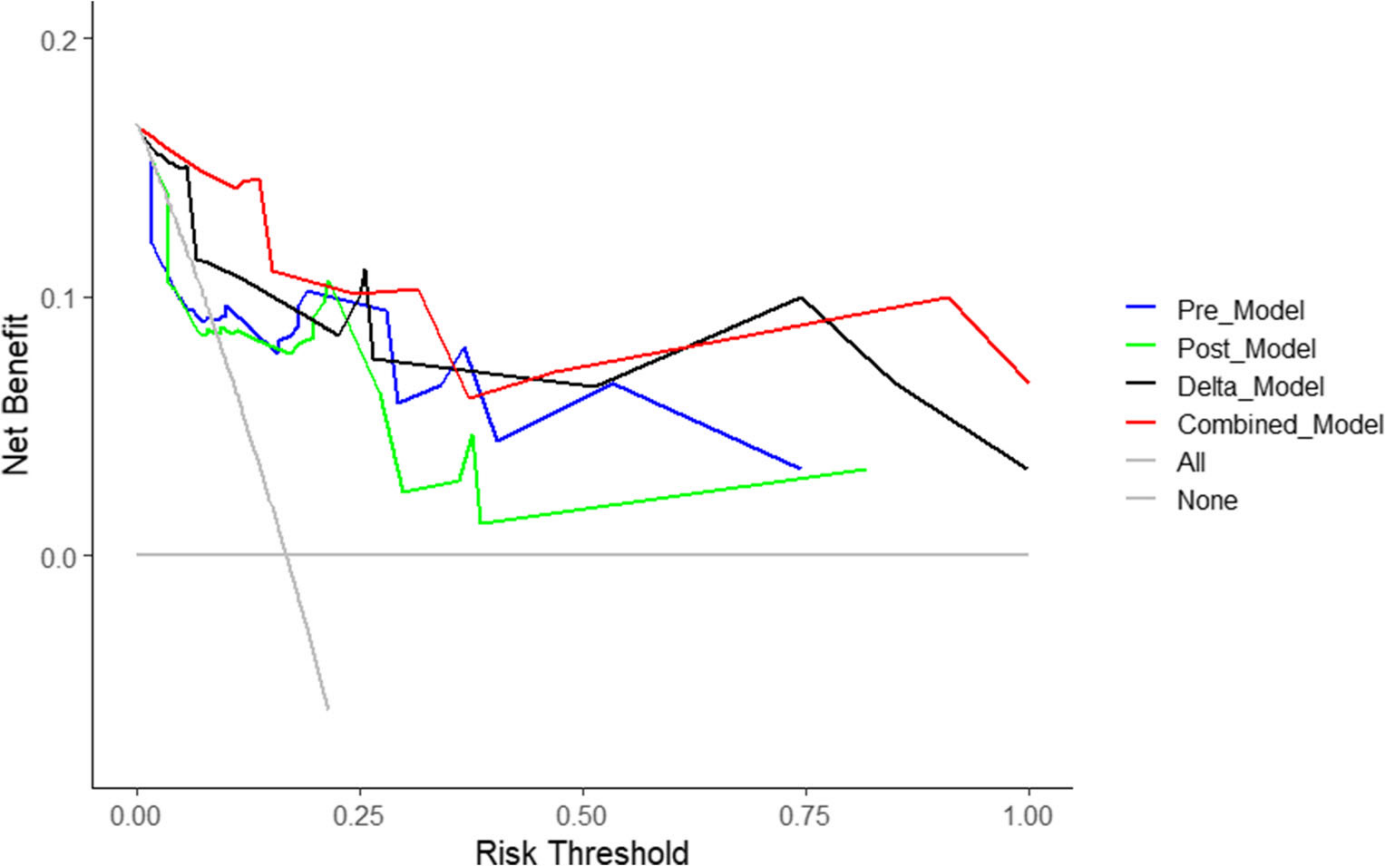
Decision curve analysis (DCA) of the four models. The net benefit is calculated by subtracting the proportion of false-positive patients from the proportion of true-positive patients, weighted by the relative harm of a false-positive result and a false-negative result. The two extreme strategies “treat all” and “treat none” are displayed as a reference. A decision model shows a clinical benefit if the decision curve shows a larger net benefit than both reference strategies

The differences in the RadScore between the pCR and N-pCR groups for each model are shown in Fig. 3. There were significant differences in the RadScore between the pCR and N-pCR groups for the Delta_Model and Combined_Model (*p* < 0.05). The performance and ROC curves of each model are shown in Table 4 and Fig. 4. Similarly, the DCA curves showed that both the Delta_Model and Combined_Model have better net returns than the None model and the All model over a wide range of risk thresholds between 0.1 and 1.0 (Fig. 5). The diagnostic performance of the Combined_Model was significantly better than that of AJCC staging and RECIST 1.1 criteria, with AUC values of 0.952 vs. 0.524 and 0.520 (*p* < 0.05), respectively. Even with the Delta_Model alone, the AUC values were also significantly higher than those of AJCC and RECIST 1.1 (0.92 vs. 0.524 and 0.520, *p* < 0.05).

**Table 4:** Multivariate logistic regression analysis

Model	OR	95%CI	*p* value	Model fit
Pre_Model				0.03
Pre_T_2__original_shape_Flatness	0.29	0.11~0.80	0.023*	
Post_Model				0.06
Post_T_1__original_shape_Flatness	0.23	0.06~0.79	0.028*	
Delta_Model				0.482
Delta_T_1__original_glcm_ClusterShade	1	1.00~1.00	0.08*	
Delta_T_2__original_glszm_SizeZoneNonUniformity	1.42	1.16~1.75	0.02*	
Combined_Model				0.998
Pre_T_2__original_shape_Flatness	2.31	0.37~14.6	0.4	
Post_T_1__original_shape_Flatness	0.23	0.03~1.83	0.2	
Delta_T_1__original_glcm_ClusterShade	1	1.00~1.00	0.014*	
Delta_T_2__original_glszm_SizeZoneNonUniformity	1.43	1.13~1.81	0.006*	

*OR*, odds ratio; *CI*, confidence interval. The goodness-of-fit of the logistic regression model was assessed using the Hosmer–Lemeshow test, and a model with *p* > 0.05 was considered to be well fitted. *p < 0.05

## Discussion

To our knowledge, this is the first study to construct efficacy prediction models based on multisequence and whole-tumor texture parameters at multiple time points (pre, post, and delta) of neoadjuvant RT and TKI for STS. The Combined_Model, in which all texture parameters of all time points were incorporated, had the highest diagnostic performance (AUC 0.952), followed by the Delta_Model (AUC 0.92), compared to routinely used RECIST 1.1 criteria and AJCC staging. Multisequence whole-tumor texture analysis can better predict the pCR results of neoadjuvant RT and TKI for STS.

We primarily focused on the early response prediction of STS patients receiving neoadjuvant RT and TKI. In the modern era, TKIs have played an emerging role in the treatment of STSs as radio-sensitizing agents [4–7]. Although previously published papers focused on the radiomics-based efficacy prediction of neoadjuvant RT and/or chemotherapy [15, 16], it remains unknown whether there is a difference with the addition of targeted therapy. It is important to screen tumors that are more sensitive to this combined treatment modality because the addition of TKIs will add some moderate toxicities, which means more harm than benefit for insensitive patients; furthermore, some patients may need chemotherapy earlier. Our study showed that patients with certain MR features are highly likely to achieve pCR after preoperative RT plus TKI, which can possibly help guide personalized treatment in the future.

This study included three sequences (T_1_FSGd, T_2_FS, and DWI) and ADC maps to predict the efficacy of neoadjuvant RT and TKI for STS. Gao et al [32] predicted the efficacy of neoadjuvant RT in 30 patients by using the radiomics features of ADC maps [32]. Crombé et al [29] analyzed 65 patients treated with neoadjuvant chemotherapy by using MRI (T_2_WI) before and after treatment. The sequences in Peeken’s study [17] on 161 sarcoma patients were also T_1_FSGd and T_2_FS [17]. In contrast to the above studies, the sequences in our study were more complete and further reduced the differences caused by inconsistent machine models. At the same time, the features finally included in our model construction were all from T_1_FSGd and T_2_FS sequences, similar to the results of Peeken et al’s [17] study. It is worth noting that the texture features corresponding to DWI and ADC were excluded in the feature screening process, which undercuts the argument that the research sequences were more complete than those in previous research. Although the field strength of the MRI scanner in our study (3.0 T) was better than that of Gao’s et al [32] study (0.35 T), the features from DWI and ADC were still slightly inferior in predicting the efficacy of STS and thus were not included for model construction. This may be related to the low signal-to-noise ratio (SNR) of DWI and ADC images [33]. In the future, the application of functional MRI (IVIM, DKI, etc.) or deep learning studies may help improve the prediction accuracy of diagnostic models for neoadjuvant RT of STS.

The Combined_Model in our study, which incorporated the image textures at all time points, had the best diagnostic performance (AUC 0.952), and the Delta_Model also had good performance (AUC 0.92). Compared with that of Peeken et al [17] (AUC of 0.75), that of Crombé et al [29] (AUC of 0.86), and that of Gao et al [32] (AUC of 0.91), the model in our study performed better for the following reasons: we used image textures at multiple time points to better reflect the changes in tumor morphology and heterogeneity and our patients were enrolled prospectively with all MR images acquired on one machine. In terms of specific texture features, there were three texture features that were finally included in our model, among which Flatness and SizeZoneNonUniformity were two texture features also included in the model of Peeken et al [17] (Delta-T_1_FSGd, Delta-T_2_ FS), and the Flatness texture feature was also statistically significant in Crombé’s study (*p* < 0.05). Flatness shows the relationship between the largest and smallest principal components in the ROI shape. SizeZoneNonUniformity measures the variability of size zone volumes in the image, with a lower value indicating more homogeneity in size zone volumes. Both these features reflect tumor heterogeneity in morphology, while another texture parameter in our study, ClusterShade, reflects the stability of the tumor grayscale. Although we focused on different neoadjuvant therapies for STSs, it seems that there was some consistency in the efficacy prediction by texture features. We also discuss some advances in the clinical and pathological evaluation of neoadjuvant therapy for STS, as detailed in the Supplementary Materials.

This study also has some limitations. First, this study is a single-center study with a relatively small number of patients, so a larger sample size is needed to further refine or validate the model, and with different machines for more generalizability would be the next step. Second, only 3 patients received RT alone due to consent withdrawal, but TKI was used as an RT-sensitizing drug in this prospective study, so we still enrolled them in this study. Our research is the first to report the early response evaluation of neoadjuvant RT and TKI; thus, it is still informative. Finally, the small sample size inevitably leads to the risk of model overfitting, and we demonstrate in the Supplementary Material that other selection or model construction methods are available for reference. Meanwhile, feature extraction requires manual or semiautomatic tumor contouring, and the results may therefore be partially biased, although two radiologists contoured the ROIs independently and another senior radiologist reviewed them.

## Conclusions

Our study shows that multisequence whole-tumor texture analysis based on MRI can well predict pCR status after neoadjuvant RT plus targeted therapy in patients with STS compared with the RECIST 1.1 criteria and AJCC staging. The combined prediction model with features at multiple time points showed the best prediction effect, followed by the Delta_Model. The prediction of pCR may help clinicians individualize clinical treatment strategies in the future. Further large-scale studies and model validation are needed to translate our findings into clinical practice.

## Supplementary information

ESM 1 (DOCX 88 kb)

## Abbreviations

ADC: Apparent diffusion coefficient
AJCC: American Joint Committee on Cancer
AUC: Area under the receiver operating characteristic curve
CI: Confidence interval
CNN: Convolutional neural network
DCA: Decision curve analysis
DWI: Diffusion-weighted imaging
FNCLCC: French Federation of Cancer Centers Sarcoma Group
GLCM: Gray level co-occurrence matrix
GLDM: Gray level dependence matrix
GLRLM: Gray level run length matrix
GLSZM: Gray level size zone matrix
ICC: Intraclass correlation coefficient
MDT: Multidisciplinary treatment
MRI: Magnetic resonance imaging
N-pCR: Non-pathological complete response
OR: Odds ratio
PCC: Pearson correlation coefficient
pCR: Pathological complete response
RECIST: Response Evaluation Criteria in Solid Tumors
ROC: Receiver operating characteristic
RT: Radiotherapy
STS: Soft tissue sarcoma
T_1_FSGd: T1-weighted with fat saturation and contrast enhancement
T_2_FS: T2-weighted with fat saturation

## Declarations

### Guarantor

The scientific guarantor of this publication is NingNing Lu (MD, Department of Radiation Oncology) and Meng Li (MD, Department of Radiology), National Cancer Center/National Clinical Research Center for Cancer/Cancer Hospital, Chinese Academy of Medical Sciences and Peking Union Medical College, Beijing, 100021, China.

### Statistics and biometry

YanMei Wang kindly provided statistical advice for this manuscript.

### Methodology

• Prospective

• Diagnostic or prognostic study

• Performed at one institution
