# Supplementary material for: Predicting pathological complete response of neoadjuvant radiotherapy and targeted therapy for soft tissue sarcoma by whole-tumor texture analysis of multisequence MRI imaging
Source: Eur Radiol. 2022 Dec 29;33(6):3984–94. doi: 10.1007/s00330-022-09362-6 (PMC10182155; doi:10.1007/s00330-022-09362-6)
Supplement: Supplementary file 1 — (DOCX 88 kb) [file 330_2022_9362_MOESM1_ESM.docx]

**Supplementary Material**

**Catalog:**

**Page2: Treatment and clinical efficacy assessment**

**Page3-4: Discussion about advances in clinical and pathological evaluation of neoadjuvant therapy for soft tissue sarcoma**

**Page5: Table S1 MRI Parameters.**

**Page6: Table S2. Pearson correlation of Pre-features.**

**Page7: Table S3. Pearson correlation of Post-features.**

**Page8: Table S4. Pearson correlation of Delta-features.**

**Page9: Discussion on the overfitting problem of the model.**

***Treatment and clinical efficacy assessment***

All patients included in this study received neoadjuvant RT with a total dose of 50 Gy in 25 fractions, with concurrent and sequential anlotinib (12 mg/day for 2 weeks every 3 weeks, with a total of 3 cycles) or apatinib (500 mg/day, 5 days per week, started from 2 weeks before RT to 4 weeks after RT) treatment, followed by planned limb-conserving wide resection (1-2 weeks apart from anlotinib or apatinib and 4-8 weeks apart from RT).

All postoperative resected specimens were reviewed and evaluated by a senior pathologist with 19 years of experience in STS diagnosis who was blinded to the clinical and MRI data. pCR was defined as less than 5% residual tumor cells as per postoperative pathology. Clinical characteristics, including age, sex, tumor region, tumor size, T stage, grade, and AJCC stage, were obtained for all patients. Grading was evaluated according to the French Federation of Cancer Centers Sarcoma Group (FNCLCC) grading system. T stage and AJCC stage were evaluated according to the 8^th^ edition of the AJCC Cancer Staging Manual. The RECIST 1.1 criteria were used to compare the tumor size changes. Informed consent was obtained before treatment.

***Discussion about advances in clinical and pathological evaluation of neoadjuvant therapy for soft tissue sarcoma***

Clinically, RECIST 1.1 is often used to evaluate the efficacy of neoadjuvant therapy for solid tumors but is probably insufficient for STS, especially to predict the pCR status. After neoadjuvant therapy for STS, it is common for the tumor to appear cystic or haemorrhagic, sometimes leading to pseudoprogression [1-3], and there is no evidence that the 30% tumor reduction in RECIST 1.1 is a truly good surrogate for the response to neoadjuvant therapy for sarcoma [4]. In the modern era, with the introduction of different RT sensitization drugs in clinical use, new methods should be investigated to predict clinical efficacy. On this basis, some scholars proposed the Choi standard based on CT images [5], and soon after, Stacchiotti et al. [6] proposed the Choi standard based on MRI images to judge via the signal intensity. Some follow-up studies also indicated that this standard was more accurate [7-9]. The Choi criteria have not been widely used in clinical practice, and more cases are needed for verification in the future. Compared with RECIST 1.1, the multi-time point prediction models established in our study have better performance. In the research of Peeken et al. [10], multiple models established by machine learning also had higher diagnostic performance than RECIST 1.1.

The pathological evaluation criteria after neoadjuvant therapy for STS are diverse, and no consensus has been reached on pathology thus far. Consistent with the studies of Bonvalot et al. [11], Soldatos et al. [12] and Peeken et al. [10], our study also selected pCR as a predictor of clinical outcomes, defined as less than 5% of residual tumor cells by postoperative pathology. In previous studies, the most widely accepted pathological assessment method was proposed by the EORTC-STBSG working group. The pathological response was graded by the different proportions of residual cells. Grades A to E reflect the number of residual tumor cells. A was defined as cell-free residual, and E was defined as more than 50% residual tumor cells [13]. Our study strictly followed this guideline in the process of pathology sampling and evaluation. In a number of studies [9, 14-16], less than 10% of residual tumor cells was defined as a better posttreatment response. Other pathological evaluation criteria are mostly based on the internal tumor necrosis rate. Some studies believe that 95% of the necrotic components in the tumor are significantly correlated with tumor prognosis, especially for studies focusing on rhabdomyosarcoma. The studies of Canter et al. [17] and Gao et al. [18] used the necrosis rate to reflect the efficacy, but this standard is controversial for STS, and some studies suggest that the necrotic component does not reflect the biological behaviour of STSs [4]. Thus, we still adopted pCR as our endpoint, since some studies have confirmed that pCR can be used as a surrogate outcome of clinical prognosis in prospective studies [11].

**Table S1 MRI Parameters.**

| **Protocol sequence** | **Slice thickness(mm)** | **Spacing** | **TR (ms)** | **TE (ms)** | **Frequency** | **Phase** | **Nex** | **ETL** | **RF Angle** | **Bandwidth(kHz)** |
| --- | --- | --- | --- | --- | --- | --- | --- | --- | --- | --- |
| Ax T_2_FS | 5 | 0 | Auto | 83.8 | 384 | NA | 3 | 28 | 110 | 100 |
| Ax DWI(b=0,1000) | 5 | 0 | Auto | Minimum | 96 | 128 | 4 | NA | NA | 250 |
| Ax T_1_FSGd | 5 | NA | 12.9 | Minimum | 320 | 320 | 1 | NA | 12 | 31.25 |

**Table S2. Pearson correlation of Pre-features.**

|  | Pre_T2_original_glrlm_GrayLevelVariance | Pre_T2_original_shape_Flatness | Pre_T2_original_firstorder_Range | Pre_T2_original_firstorder_Kurtosis |
| --- | --- | --- | --- | --- |
| Pre_T2_original_glrlm_GrayLevelVariance | 1.00 | 0.06 | 0.81* | 0.30 |
| Pre_T2_original_shape_Flatness | 0.06 | 1.00 | -0.24 | -0.27 |
| Pre_T2_original_firstorder_Range | 0.81* | -0.24 | 1.00 | 0.64 |
| Pre_T2_original_firstorder_Kurtosis | 0.3 | -0.27 | 0.64 | 1.00 |

**Table S3. Pearson correlation of Post-features.**

|  | Post_T1_original_shape_Flatness | Post_ADC_original_gldm_HighGrayLevelEmphasis |
| --- | --- | --- |
| Post_T1_original_shape_Flatness | 1.00 | -0.19 |
| Post_ADC_original_gldm_HighGrayLevelEmphasis | -0.19 | 1.00 |

**Table S4. Pearson correlation of Delta-features.**

|  | Delta_T2_original_glszm_SizeZoneNonUniformity | Delta_T2_original_firstorder_Energy | Delta_T2_original_firstorder_Range | Delta_T2_original_firstorder_TotalEnergy | Delta_T1_original_glcm_ClusterShade | Delta_T1_original_shape_Sphericity |
| --- | --- | --- | --- | --- | --- | --- |
| Delta_T2_original_glszm_SizeZoneNonUniformity | 1.00 | 0.55 | 0.52 | 0.38 | 0.05 | 0.08 |
| Delta_T2_original_firstorder_Energy | 0.55 | 1.00 | 0.43 | 0.92* | 0.00 | -0.09 |
| Delta_T2_original_firstorder_Range | 0.52 | 0.43 | 1.00 | 0.44 | 0.09 | 0.04 |
| Delta_T2_original_firstorder_TotalEnergy | 0.38 | 0.92* | 0.44 | 1.00 | 0.01 | -0.12 |
| Delta_T1_original_glcm_ClusterShade | 0.05 | 0.00 | 0.09 | 0.01 | 1.00 | 0.21 |
| Delta_T1_original_shape_Sphericity | 0.08 | -0.09 | 0.04 | -0.12 | 0.21 | 1.00 |

***Discussion on the overfitting problem of the model.***

we reselected and remodeled the original data, including the following three methods: **Method 1.** Establishment of Random Forest and AdaBoost models based on the original filtered data; **Method 2.** Changes in the feature selection method, gradually reducing the dimensions and establishing three models of logistic regression, random forest and AdaBoost through the variance method, Pearson correlation test and LASSO; **Method 3**. Based on the first two steps of the original feature selection method (t test, Pearson correlation test), the addition of LASSO to further selection.

**Method 1**. Random forest and AdaBoost models were established based on the original filtered data.

First, the original filtered data are as follows:

| Pre | T2_original_shape_Flatness |
| --- | --- |
| Post | T1_original_shape_Flatness |
| Delta | T1_original_glcm_ClusterShade, T2_original_glszm_SizeZoneNonUniformity |

The performance of the models is shown as follows:

Table 1: Random Forest Model performance for predicting pCR status after neoadjuvant RT and targeted therapy in patients with STS.

|  | Pre-Model | Post-Model | Delta-Model | Combined-Model |
| --- | --- | --- | --- | --- |
| AUC | 0.948 | 0.944 | 0.952 | 1.0 |
| 95% CI | 0.868~1.0 | 0.864~1.0 | 0.893~1.0 | 1.0~1.0 |
| Sensitivity | 0.6 | 0.6 | 0.4 | 1 |
| Specificity | 1 | 0.96 | 1 | 1 |
| Accuracy | 0.933 | 0.9 | 0.9 | 0.933 |

Table 2: AdaBoost model performance for predicting pCR status after neoadjuvant RT and targeted therapy in patients with STS.

|  | Pre-Model | Post-Model | Delta-Model | Combined-Model |
| --- | --- | --- | --- | --- |
| AUC | 0.944 | 0.968 | 0.964 | 1.0 |
| 95% CI | 0.84~1.0 | 0.905~1.0 | 0.897~1.0 | 1.0~1.0 |
| Sensitivity | 0.8 | 0.6 | 0.8 | 0.8 |
| Specificity | 0.92 | 1 | 1 | 1 |
| Accuracy | 0.9 | 0.933 | 0.967 | 0.967 |

**Method 2.** Change the feature selection method, gradually reduce the dimensions, and establish three models of logistic regression, random forest and AdaBoost through the variance method, Pearson correlation test and LASSO.

First, the final features changed after selection by the above methods:

| Pre | T2_original_shape_Flatness, T2_original_firstorder_Range |
| --- | --- |
| Post | ADC_original_glcm_ClusterShade, T1_original_shape_Flatness |
| Delta | T1_original_firstorder_Skewness, T2_original_glcm_ClusterShade, T2_original_glszm_SizeZoneNonUniformity |

Pre, post, and delta all have more features than before. Let us look at the situation after the model is built:

Table 3: Three pre-model performance outcomes for predicting pCR status after neoadjuvant RT and targeted therapy in patients with STS.

|  | Logistic Regression Model | Random Forest Model | AdaBoost Model |
| --- | --- | --- | --- |
| AUC | 0.848 | 0.936 | 0.952 |
| 95% CI | 0.597~1.0 | 0.815~1.0 | 0.872~1.0 |
| Sensitivity | 0.6 | 0.4 | 0.6 |
| Specificity | 0.96 | 1 | 0.96 |
| Accuracy | 0.9 | 0.9 | 0.9 |

Table 4: Three post-model performance outcomes for predicting pCR status after neoadjuvant RT and targeted therapy in patients with STS.

|  | Logistic Regression Model | Random Forest Model | AdaBoost Model |
| --- | --- | --- | --- |
| AUC | 0.848 | 0.884 | 0.908 |
| 95% CI | 0.683~0.981 | 0.769~0.973 | 0.808~0.983 |
| Sensitivity | 0.6 | 0.2 | 0.4 |
| Specificity | 0.8 | 1 | 0.96 |
| Accuracy | 0.767 | 0.867 | 0.867 |

Table 5: Three Delta-Model performance outcomes for predicting pCR status after neoadjuvant RT and targeted therapy in patients with STS.

|  | Logistic Regression Model | Random Forest Model | AdaBoost Model |
| --- | --- | --- | --- |
| AUC | 0.92 | 0.896 | 0.976 |
| 95% CI | 0.768~1.0 | 0.696~1.0 | 0.92~1.0 |
| Sensitivity | 0.6 | 0.6 | 0.8 |
| Specificity | 1 | 1 | 1 |
| Accuracy | 0.933 | 0.933 | 0.967 |

Table 6: Three Combined-Model performances for predicting pCR status after neoadjuvant RT and targeted therapy in patients with STS.

|  | Logistic Regression Model | Random Forest Model | AdaBoost Model |
| --- | --- | --- | --- |
| AUC | 1.0 | 0.952 | 0.952 |
| 95% CI | 1.0~1.0 | 0.864~1.0 | 0.846~1.0 |
| Sensitivity | 0.8 | 0.6 | 0.8 |
| Specificity | 1 | 1 | 1 |
| Accuracy | 0.967 | 0.933 | 0.967 |

**Method 3.** Based on the first two steps of the original feature selection method (t test, Pearson correlation test), LASSO was added for further selection.

The feature selected by this method is the same as the feature selected by the original research method (t test, Pearson correlation test, and logistic), and the results of the established model are also consistent. Therefore, the details are not shown here.

After recalculation through the above three methods, our team is more inclined to keep the original feature selection and model building methods for the following reasons:

1. In this small sample size study, the risk of overfitting is difficult to avoid. Usually, the methods to solve overfitting include (1) increasing the sample size, (2) reducing the number of features, and (3) changing the model.

First, it is difficult to further increase the sample size in our study due to the low incidence rate of STS and the relatively long time from enrolment to surgery. In previous efficacy evaluation studies related to STS, the sample sizes were not large. For example, there were only 23 patients in the article published by Theodoros Soldatos [12] in “Radiology”. The inclusion of more patients is the direction of our future efforts. Second, we tried to reduce the number of features. We changed the feature selection method in **method 2**, but more features were finally included in the model construction, which still had the risk of overfitting. The AUC of the combined model (logistic regression) was 1, and the model showed overfitting, which was not desirable. Finally, we changed the model. **Method 1** and **method 2** were both used to build the random forest and AdaBoost models, but **method 1** showed overfitting in the combined model (AUC=1), so **method 1** was not desirable. The model built by **method 2** was not much better than our original logical regression model, and the logical regression model is more interpretable and easier to implement and understand. Moreover, the most common disadvantage of the logistic regression model is underfitting instead of overfitting. The random forest model construction method can indeed reduce the risk of overfitting, but it is most commonly used for multivariate classification variables. The amount of data required is large, and the model is not highly interpretable. The AdaBoost algorithm can indeed reduce the risk of overfitting, but it is vulnerable to noise interference, and imbalanced data easily lead to a decline in classification accuracy.

2. The texture analysis method used in this study is a traditional method, and many published articles have also used this method [19-21]. However, if meaningful features are selected through texture analysis, it is also instructive or suggestive to try to build a model. In the future, we will include more patients on this basis and conduct better and more complete research.

3. Our original analysis method added the Hosmer–Lemeshow test to assess the goodness-of-fit of the models (p>0.05). Although the risk of overfitting cannot be completely excluded from model fit analysis, it can also show that our model is well fitted.

**References:**

1. Tanaka K, Ogawa G, Mizusawa J et al (2018) Prospective comparison of various radiological response criteria and pathological response to preoperative chemotherapy and survival in operable high-grade soft tissue sarcomas in the Japan Clinical Oncology Group study JCOG0304. World J Surg Oncol 16:162

2. Roberge D, Skamene T, Nahal A, Turcotte RE, Powell T, Freeman C (2010) Radiological and pathological response following pre-operative radiotherapy for soft-tissue sarcoma. Radiother Oncol 97:404-7

3. Rothermundt C, Fischer GF, Bauer S et al (2018) Pre- and Postoperative Chemotherapy in Localized Extremity Soft Tissue Sarcoma: A European Organization for Research and Treatment of Cancer Expert Survey. Oncologist 23:461-467

4. Gennaro N, Reijers S, Bruining A et al (2021) Imaging response evaluation after neoadjuvant treatment in soft tissue sarcomas: Where do we stand? Crit Rev Oncol Hematol 160:103309

5. Choi H, Charnsangavej C, Faria SC et al (2007) Correlation of computed tomography and positron emission tomography in patients with metastatic gastrointestinal stromal tumor treated at a single institution with imatinib mesylate: proposal of new computed tomography response criteria. J Clin Oncol 25:1753-9

6. Stacchiotti S, Verderio P, Messina A et al (2012) Tumor response assessment by modified Choi criteria in localized high-risk soft tissue sarcoma treated with chemotherapy. Cancer 118:5857-66

7. Stacchiotti S, Collini P, Messina A et al (2009) High-grade soft-tissue sarcomas: tumor response assessment--pilot study to assess the correlation between radiologic and pathologic response by using RECIST and Choi criteria. Radiology 251:447-56

8. Esser M, Kloth C, Thaiss WM et al (2018) CT-response patterns and the role of CT-textural features in inoperable abdominal/retroperitoneal soft tissue sarcomas treated with trabectedin. Eur J Radiol 107:175-182

9. Crombé A, Le Loarer F, Cornelis F et al (2019) High-grade soft-tissue sarcoma: optimizing injection improves MRI evaluation of tumor response. Eur Radiol 29:545-555

10. Peeken JC, Asadpour R, Specht K et al (2021) MRI-based delta-radiomics predicts pathologic complete response in high-grade soft-tissue sarcoma patients treated with neoadjuvant therapy. Radiother Oncol 164:73-82

11. Bonvalot S, Rutkowski PL, Thariat J et al (2019) NBTXR3, a first-in-class radioenhancer hafnium oxide nanoparticle, plus radiotherapy versus radiotherapy alone in patients with locally advanced soft-tissue sarcoma (Act.In.Sarc): a multicentre, phase 2-3, randomised, controlled trial. Lancet Oncol 20:1148-1159

12. Soldatos T, Ahlawat S, Montgomery E, Chalian M, Jacobs MA, Fayad LM (2016) Multiparametric Assessment of Treatment Response in High-Grade Soft-Tissue Sarcomas with Anatomic and Functional MR Imaging Sequences. Radiology 278:831-40

13. Wardelmann E, Haas RL, Bovée JV et al (2016) Evaluation of response after neoadjuvant treatment in soft tissue sarcomas; the European Organization for Research and Treatment of Cancer-Soft Tissue and Bone Sarcoma Group (EORTC-STBSG) recommendations for pathological examination and reporting. Eur J Cancer 53:84-95

14. Crombe A, Perier C, Kind M et al (2019) T2 -based MRI Delta-radiomics improve response prediction in soft-tissue sarcomas treated by neoadjuvant chemotherapy. J Magn Reson Imaging 50:497-510

15. Crombé A, Fadli D, Buy X, Italiano A, Saut O, Kind M (2020) High-Grade Soft-Tissue Sarcomas: Can Optimizing Dynamic Contrast-Enhanced MRI Postprocessing Improve Prognostic Radiomics Models? J Magn Reson Imaging 52:282-297

16. Crombé A, Le Loarer F, Stoeckle E et al (2018) MRI assessment of surrounding tissues in soft-tissue sarcoma during neoadjuvant chemotherapy can help predicting response and prognosis. Eur J Radiol 109:178-187

17. Canter RJ, Martinez SR, Tamurian RM et al (2010) Radiographic and histologic response to neoadjuvant radiotherapy in patients with soft tissue sarcoma. Ann Surg Oncol 17:2578-84

18. Gao Y, Kalbasi A, Hsu W et al (2020) Treatment effect prediction for sarcoma patients treated with preoperative radiotherapy using radiomics features from longitudinal diffusion-weighted MRIs. Phys Med Biol 65:175006

19. Zhang Q, Yu X, Ouyang H et al (2021) Whole-tumor texture model based on diffusion kurtosis imaging for assessing cervical cancer: a preliminary study. Eur Radiol 31:5576-5585

20. Zhao L, Liang M, Yang Y, Xie L, Zhang H, Zhao X (2022) The added value of full and reduced field-of-view apparent diffusion coefficient maps for the evaluation of extramural venous invasion in rectal cancer. Abdom Radiol (NY) 47:48-55

21. Zhao L, Liang M, Yang Y, Zhao X, Zhang H (2021) Histogram models based on intravoxel incoherent motion diffusion-weighted imaging to predict nodal staging of rectal cancer. Eur J Radiol 142:109869
